# Consistent multi-level trophic effects of marine reserve protection across northern New Zealand

**DOI:** 10.1371/journal.pone.0177216

**Published:** 2017-05-24

**Authors:** Graham J. Edgar, Rick D. Stuart-Smith, Russell J. Thomson, Debbie J. Freeman

**Affiliations:** 1 Institute of Marine and Antarctic Studies, Hobart, Tasmania, Australia; 2 Centre for Research in Mathematics, Western Sydney University, Parramatta Campus, Penrith, New South Wales, Australia; 3 Department of Conservation—*Te Papa Atawhai*, Wellington, New Zealand; Department of Agriculture and Water Resources, AUSTRALIA

## Abstract

Through systematic Reef Life Survey censuses of rocky reef fishes, invertebrates and macroalgae at eight marine reserves across northern New Zealand and the Kermadec Islands, we investigated whether a system of no-take marine reserves generates consistent biodiversity outcomes. Ecological responses of reef assemblages to protection from fishing, including potential trophic cascades, were assessed using a control-impact design for the six marine reserves studied with associated reference sites, and also by comparing observations at reserve sites with predictions from random forest models that assume reserve locations are fished. Reserve sites were characterised by higher abundance and biomass of large fishes than fished sites, most notably for snapper *Chrysophrys auratus*, with forty-fold higher observed biomass inside relative to out. In agreement with conceptual models, significant reserve effects not only reflected direct interactions between fishing and targeted species (higher large fish biomass; higher snapper and lobster abundance), but also second order interactions (lower urchin abundance), third order interactions (higher kelp cover), and fourth order interactions (lower understory algal cover). Unexpectedly, we also found: (i) a consistent trend for higher (~20%) *Ecklonia* cover across reserves relative to nearby fished sites regardless of lobster and urchin density, (ii) an inconsistent response of crustose coralline algae to urchin density, (iii) low cover of other understory algae in marine reserves with few urchins, and (iv) more variable fish and benthic invertebrate communities at reserve relative to fished locations. Overall, reef food webs showed complex but consistent responses to protection from fishing in well-enforced temperate New Zealand marine reserves. The small proportion of the northeastern New Zealand coastal zone located within marine reserves (~0.2%) encompassed a disproportionately large representation of the full range of fish and benthic invertebrate biodiversity within this region.

## Introduction

New Zealand has played a key role in the development of marine protected areas (MPAs) worldwide. It was amongst the first countries to establish a no-fishing MPA (i.e. a ‘marine reserve’), with the Cape Rodney to Okakari Point Marine Reserve declared in 1975. It was also arguably the first country to recognise a critical need for protection of its biodiversity heritage through the establishment of a network of marine reserves, a process facilitated by the *Marine Reserves Act* 1971 [1–3], and more recently the New Zealand *Marine Protected Areas Policy and Implementation Plan* [4].

Under the Marine Reserves Act 1971, New Zealand’s marine reserves have a primary purpose of providing for scientific research, but are also recognised for the important role they play in protecting the range of marine biodiversity in New Zealand’s waters [4, 5]. Public benefits generated by marine reserve networks have been suggested to include: (i) safeguarding representative examples of local marine biodiversity for future generations, (ii) providing reference sites for scientific research that are relatively free from human impacts, (iii) augmenting opportunities for non-extractive recreational and educational activities, and (iv) providing insurance against fishery stock collapse during an era of changing climate when traditional fisheries management models are set in a context exceeding known environmental bounds [6].

While still far from complete and small in total area (~9.8% of territorial seas, when large reserves at the Kermadec Islands and the subantarctic islands are included), the New Zealand marine reserve system currently encompasses 44 marine reserves and is amongst the largest marine reserve networks worldwide. In addition to marine reserves, several other types of protected areas are recognised in New Zealand as MPAs for the purposes of MPA planning, including some fisheries management zones and cable protection zones [7].

The MPA model currently applied in New Zealand is, however, unusual in a global context, with a focus on small no-fishing marine reserves rather than large multi-zoned marine parks, as are commonly applied, for example, across Australia. Within the Australian system, small no-fishing areas are typically interspersed within larger ‘habitat protection zones’ utilised by recreational and in some cases commercial fishers (e.g., the Great Barrier Reef and Lord Howe Island Marine Parks). Regulations within habitat protection zones within marine parks vary greatly between jurisdictions, ranging from open access zones that allow all conventional forms of fishing to line angling from beaches only.

Improved understanding of the ecological benefits of different MPA management models requires observations at a range of locations where protected zones have been established. Outcomes of single MPA studies are difficult to generalise because of the likelihood of locally-idiosyncratic responses. Ecological patterns in MPAs can be driven by novel interactions involving socio-ecological and governance factors (including age, size, location, type of regulation, pre-existing fishing effort, level of enforcement, and extent of community support) [8–10].

Ecological responses are further complicated by the intricacy of interactions amongst marine species [11]. Removal of fishing pressure, with consequent recovery of large predatory species that were formerly targeted by fishers, affects the food web at various trophic levels. Thus, fishing impacts ripple synergistically and antagonistically across ecosystems, resulting in biological communities and habitats that can change over time and differ markedly from unfished areas [12, 13]. Fishing affects temperate reef food webs in at least four ways: (i) direct removal of targeted species, (ii) increase in numbers of the prey of targeted species once predators are removed, (iii) decrease in abundance of macroalgae and small animals consumed by the prey of targeted species, and (iv) changes in abundance of organisms affected by decreased algal cover. Seminal studies demonstrating such effects have largely been undertaken within New Zealand marine reserves [12, 14–17], where prohibitions on fishing have resulted in: (i) increased populations of exploited species such as snapper and rock lobsters, (ii) decreased urchin numbers following increased predation pressure from these predators, (iii) transformation of urchin barrens to kelp forests following reduction in urchin grazing pressure, and (iv) increased numbers of amphipods and other organisms associated with kelp. Such trends are, however, not universal across the New Zealand marine reserve system [11], and some have rarely been observed elsewhere.

The present study was initiated with the primary aim of assessing ecological differences across a range of New Zealand marine reserves compared to fished coastlines, ultimately to allow more informed predictions when assessing benefits and costs associated with expanding MPA networks, particularly networks of small no-take marine reserves.

## Materials and methods

### Ethics statement

Permission for fieldwork, including the Kermadec Islands, was granted from the Department of Conservation, New Zealand (DOCDM-207148). Field studies were observational, and did not involve manipulation of any endangered or protected species.

### Sites studied

**Fig 1:**
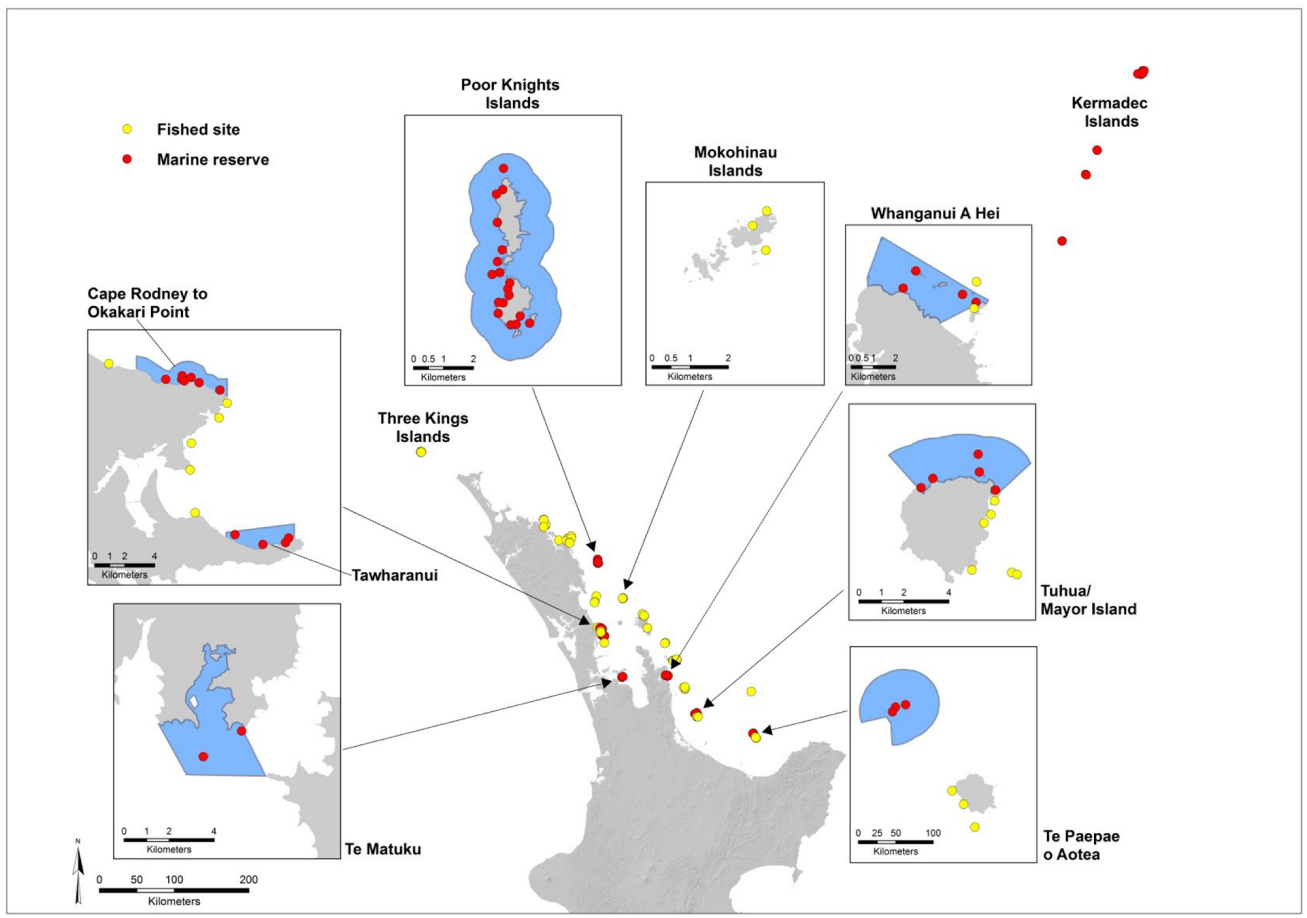
Map of New Zealand sites surveyed by Reef Life Survey (RLS) divers. Note that overlapping sites are hidden (N = 123). The map of sites can be expanded and explored on the RLS website (http://reeflifesurvey.com).

Underwater visual surveys of fishes, mobile invertebrates and sessile biota were undertaken along the North Island of New Zealand and at the Kermadec Islands using Reef Life Survey methodology [18] from 30 Sep 2012 to 1 Jan 2013 (Fig 1). Additional data from 12 sites in the Poor Knights Islands and Cape Rodney to Okakari Point reserves, which had been surveyed earlier by Reef Life Survey divers (mostly in 2009), were also included in analyses. Four of these sites were resurveyed during 2012–2013 surveys.

A total of 57 sites investigated were within eight marine reserves (Fig 1), including 14 off the Kermadec Islands Marine Reserve (700 km northeast of New Zealand), while 66 sites were surveyed along fished sections of the coast and associated islands. Sites in six marine reserves were matched with reference sites located nearby with similar survey depths and underwater visibility (Table 1, Fig 1). However, no suitable reference sites exist for the Kermadec Islands, nor could be found for Te Matuku Marine Reserve, a shallow turbid reserve near Auckland. Sites were distributed across three of the Marine Ecoregions of the World [19]: Kermadec Islands, Three Kings-North Cape, and North-eastern New Zealand.

**Table 1:** Number of sites surveyed in different NZ marine reserves and at nearby reference sites in the relatively turbid inshore region adjacent to the coast and at offshore islands. Mean underwater visibility and water depths are also shown.

Isolation	Marine Reserve	Zone	Sites	Visibility (m)	Depth (m)
Inshore	Cape Rodney—Okakari Point Marine Reserve	Reserve	7	5.6	7.8
		Fishing	3	4.5	6.5
	Cathedral Cove Marine Reserve	Reserve	4	7.8	10.0
		Fishing	2	8.2	7.6
	Tawharanui Marine Reserve	Reserve	4	4.3	6.9
		Fishing	2	3.5	6.5
	Te Matuku Marine Reserve	Reserve	2	1.3	3.3
	Non-protected	Fishing	18	8.8	10.3
Offshore	Kermadec Islands	Reserve	14	31.9	11.1
	Poor Knights Island Marine Reserve	Reserve	18	14.6	10.9
		Fishing	3	17.3	12.7
	Te Paepae o Aotea (Volkner Rocks) Marine Reserve	Reserve	3	14.0	11.5
		Fishing	3	14.6	8.9
	Tuhua/Mayor Island marine reserve	Reserve	5	11.0	10.3
		Fishing	3	19.5	8.4
	Non-protected	Fishing	32	15.5	9.8

### Underwater visual transect methodology

All surveys were undertaken using standardised underwater visual census methods applied globally through Reef Life Survey (RLS) [20]. These methods are summarised here, but further details can be downloaded from: http://reeflifesurvey.com. The RLS database now includes data from over 9,000 transects in 90 of the world’s ecoregions [19], 49 countries and spanning 146° latitude, in all ocean basins. Surveys were undertaken by committed volunteer divers trained by professional biologists on an individual basis to a scientific standard of data gathering. In earlier analyses, no detectable statistical differences were found between data generated by trained RLS volunteers and professional biologists [21].

Three survey components (fishes, mobile macroinvertebrates, sessile biota) were completed along the same 50 m transect lines, each line laid along a depth contour on predominantly rocky reef habitat. Across all sites, the major habitats were laminarian kelp (generally *Ecklonia radiata*: mean 32%), crustose coralline algae (generally urchin barrens: 19%), foliose macro-algae (red, green and brown algae from 5 to 20 cm in height:18%), turfing algae (fine closely cropped algae <5 cm in height: 15%) and fucoid kelp (5%). Multiple transects were usually surveyed at each site, generally parallel at different depths when the reef was sufficiently wide. Underwater visibility and depth (range 2–27 m) of the transect contour were recorded at the time of each survey, with visibility estimated along the transect line (Table 1).

All fish species sighted within 5 m blocks on either side of the transect line were recorded on waterproof paper as divers swam slowly beside the line. The number and estimated size-category of each species was also recorded. Size categories used were 25, 50, 75, 100, 125, 150, 200, 250, 300, 350, 400, 500, 625 mm, and above, which represent total fish length (from snout to tip of tail). All species sighted within the blocks were recorded, including those with unknown identity. Digital photographs were taken of unidentified animals to later confirm identities with appropriate taxonomic experts.

Large mobile macro-invertebrates (molluscs, echinoderms and crustaceans > 2.5 cm) were surveyed along the same transect lines set for fish surveys. Divers swam along the bottom, up each side of the transect line, recording all mobile macroinvertebrates on the reef surface within 1 m of the line. This required brushing aside the kelp canopy when present and searching along crevices and undercuts, but without moving rocks.

Information on the percentage cover of sessile invertebrates and macroalgae along the transect lines set for fish and invertebrate surveys were recorded using photoquadrats taken every 2.5 m along the 50 m transect. Digital photoquadrats were taken vertically-downward from a height sufficient to encompass an area of approximately 0.3 m x 0.3 m. Photoquadrats were not possible at some sites due to poor image quality or camera failure. In total, images were available for 107 of the 123 sites surveyed for fishes and benthic invertebrates.

The percentage cover of different macroalgal, coral, sponge and other attached invertebrate species was obtained from photoquadrats by using pre-defined substrate cover categories based on the ‘Collaborative and Automated Tools for Analysis of Marine Imagery’ (CATAMI) classification system [22]. The substrate was recorded under each of five points overlaid on each image, such that 100–110 points were counted for each transect. To provide a percentage cover estimate for the full transect, the number of points counted for each substrate cover category was divided by total points less undefined shadow and tape areas. The category referred to here as *Ecklonia* contains all laminarian kelps. This category included a few counts of *Lessonia variegata*; however, those records comprised a very small proportion of the total (<2%) compared to *Ecklonia radiata*.

### Statistical analyses

A range of univariate metrics were calculated from survey data: total biomass of all fishes and large fishes (> 25cm), total abundance of all fishes and large fishes, fish species richness, total biomass of four fish trophic groups (benthic carnivores, herbivores, higher carnivores, planktivores), total abundance of sea urchins (all species), abundance of lobsters (Palinuridae and Scyllaridae), and percent cover of *Ecklonia*, fucoid kelps (e.g. *Carpophyllum* spp., *Xiphophora chondrophylla*), other foliose algae (red, green and brown algae >10 cm height), turf algae (<10 cm height), and sand. The four major fish trophic groups were categorised using dietary information obtained from Fishbase (www.fishbase.org), as described by Soler et al. [23]. Higher carnivores comprised fishes with diets primarily composed of other fishes, decapods and cephalopods. Benthic carnivores fed predominantly on invertebrate fauna, most commonly peracarid crustaceans, molluscs, polychaetes, sponges or corals. Herbivorous species included detritivorous and omnivorous species. Planktivorous species consumed planktonic crustaceans as a primary food source. All metrics represent mean values per 500 m^2^ transect block for fishes, and per 50 m^2^ transect block for mobile invertebrates.

Collection of detailed data on fishes, including species-level identities, length classes and abundance information, allowed the calculation of species-specific biomass estimates. The RLS database includes coefficients for length—weight relationships obtained for each species (in some cases genus and family) from Fishbase (www.fishbase.org). When length—weight relationships were described in Fishbase in terms of standard length or fork length rather than total length, additional length-length relationships provided in Fishbase allowed conversion to total length, as estimated by divers. For improved accuracy in biomass estimates, the bias in divers’ perception of fish size underwater was additionally corrected using the mean relationship provided in Edgar et al. [24], where a consistent bias was found amongst divers that led to underestimation of small fish sizes and overestimation of large fish sizes. Note that estimates of fish abundance made by divers can be greatly affected by fish behaviour for many species [24]. Consequently, biomass determinations, like abundance estimates, can reliably be compared only in a relative sense (i.e. for comparisons with data collected using the same methods) rather than providing an accurate absolute estimate of fish biomass for a patch of reef.

The significance of marine reserve effects was assessed using univariate PERMANOVA [25]. For general reserve effect analyses, the categorical fixed factor ‘*zone*’ (two levels: marine reserve and fished) was crossed with another fixed factor ‘*location*’ (six reserves with associated reference sites; Table 1). Marine reserves at the Kermadec Islands and Te Matuku were excluded from analyses because of an absence of comparable reference locations. Means of metrics across all transects at each site were considered replicates in PERMANOVA runs. Residuals were permutated under a reduced Type III (partial) model [25]. One-tailed tests were applied for *zone* given that tests related to one-directional hypotheses (e.g. fish biomass is greater in marine reserves than fished coasts).

PERMANOVA calculations utilised a similarity matrix based on Euclidean Distance, with log (x+1) transformation applied. Analytical outputs (sum of squares, mean squares, *F*-values) were thus identical to those calculated using analysis of variance (ANOVA) other than *P*-values, which were calculated using permutation procedures rather than assumptions of a normal distribution [25]. As our interest in this study was focused on whether a significant generalised response occurred across the reserves as a set rather than responses in individual reserves (which will vary greatly due to local factors), the zone test involved an F-value calculated with *zone* x *location* as denominator. The power of this test of consistent response across reserves is much lower than if calculated on a region-wide basis using residual error, but the test is general.

Non-metric multidimensional scaling was used to visualise relationships in community structure amongst the marine reserves surveyed. Mean biomasses of different species were firstly calculated for each transect and summed, then the mean of site means determined as the basis for similarity matrices associated with ecoregional and marine reserve plots. Bray-Curtis dissimilarity and log (x+1) transformed data were applied for multivariate analyses involving fish biomass and invertebrate abundance, and Euclidian distance and non-transformed data applied for percent cover digitised from photoquadrats.

Inside/outside marine reserve comparisons were potentially confounded by idiosyncratic variability between sites surveyed, such that means associated with a group of sites within marine reserves can differ from means associated with reference locations because of natural pre-existing spatial variability that is unrelated to a reserve effect. In a related analysis of five Australian marine protected areas (MPAs) [9], effects associated with change through time within MPAs relative to outside (the protection effect) were subtle (4% of total variation) and required considerable power to distinguish, while pre-existing differences between sets of sites inside and outside the MPAs were more easily observed (8% of total variation).

Such spatial confounding has been reduced in this study by interspersion of reference sites across a system of multiple reserves, as it is unlikely that similar trends in ecological difference between reserve and reference locations occur systematically across marine reserves located tens to hundreds of kilometres apart. We additionally reduced the likelihood of spatial confounding using a different analytical framework that took into account variability in natural features to predict characteristics of marine reserves if that area was open to fishing. Thus, in addition to using the mean of data values from nearby fished sites as a reference for assessing change associated with reserves, we also calculated an alternative counterfactual mean using all fished sites surveyed and their associations with latitude, longitude, depth and seven environmental factors (Table 2).

**Table 2:** Covariate data used as predictor variables in random forest models. The index of population pressure was calculated by fitting a smoothly tapered surface to each settlement point on a year 2000 world population density grid [44] using the quadratic kernel function [45]. Populations were screened for a density greater than 1000 people per 0.04 degree cell, and the search radius was set at 3.959 degrees. BIO variables are described in [46].

Variable abbreviation	Variable	Units	Scale
BIO_phosphate	Mean phosphate	mol/Ml	5 arcmin (9.2 km)
BIO_silicate	Mean silicate	mol/Ml	5 arcmin
BIO_parmean	Photosynthetically-available radiation	Einstein/m^3^/day	5 arcmin
BIO_SST_mean	Mean sea surface temperature	°C	5 arcmin
BIO_SST_range	Range of sea surface temperature	°C	5 arcmin
POP_index	Index of population pressure	index	2.46 arcmin
Depth	Transect depth	m	
Visibility	Underwater visibility	m	
SiteLat	Site latitude	decimal degrees	0.0001°
SiteLong	Site longitude	decimal degrees	0.0001°

Models were developed using random forests [26], a machine learning protocol that predicted the distribution of total fish biomass and other community metrics for fished locations around northern New Zealand. Each random forest consisted of 2000 regression trees, where each tree was fitted to a bootstrap sample of the biological data using a recursive partitioning procedure. Random forest analyses also contain cross validation routines based on random subsets of survey sites and covariate predictors that are excluded during development of each tree (the ‘out-of-bag’ data). Cross validation using out-of-bag data allow estimation of prediction performance (R^2^).

Random forests models based on survey data obtained from 66 fished sites studied described relationships between the distribution of 10 environmental and geospatial covariates (Table 1) and seven of the univariate metrics analysed by PERMANOVA: fish biomass, large (>25 cm length) fish biomass, large fish abundance, fish species richness, *Ecklonia* cover, fucoid algal cover, and other foliose algae cover. These models were then used to predict each community metric at different transect depths at the 41 marine reserve sites investigated in the six reserves of primary interest.

Protection effects for fish data were calculated for each transect as the difference between values observed and values predicted if the site was fished, using the log ratio of observed/predicted value (e.g., log(m/p), where m is measured value and p is value predicted if the site was fished). Effect size was calculated as the difference between observed and predicted values for photoquadrat cover data. Because of numerous zero values, random forest models could not be developed for rock lobster density, urchin density, crustose coralline algal cover, or turf algal cover.

## Results

### Faunal and floral community patterns

**Fig 2:**
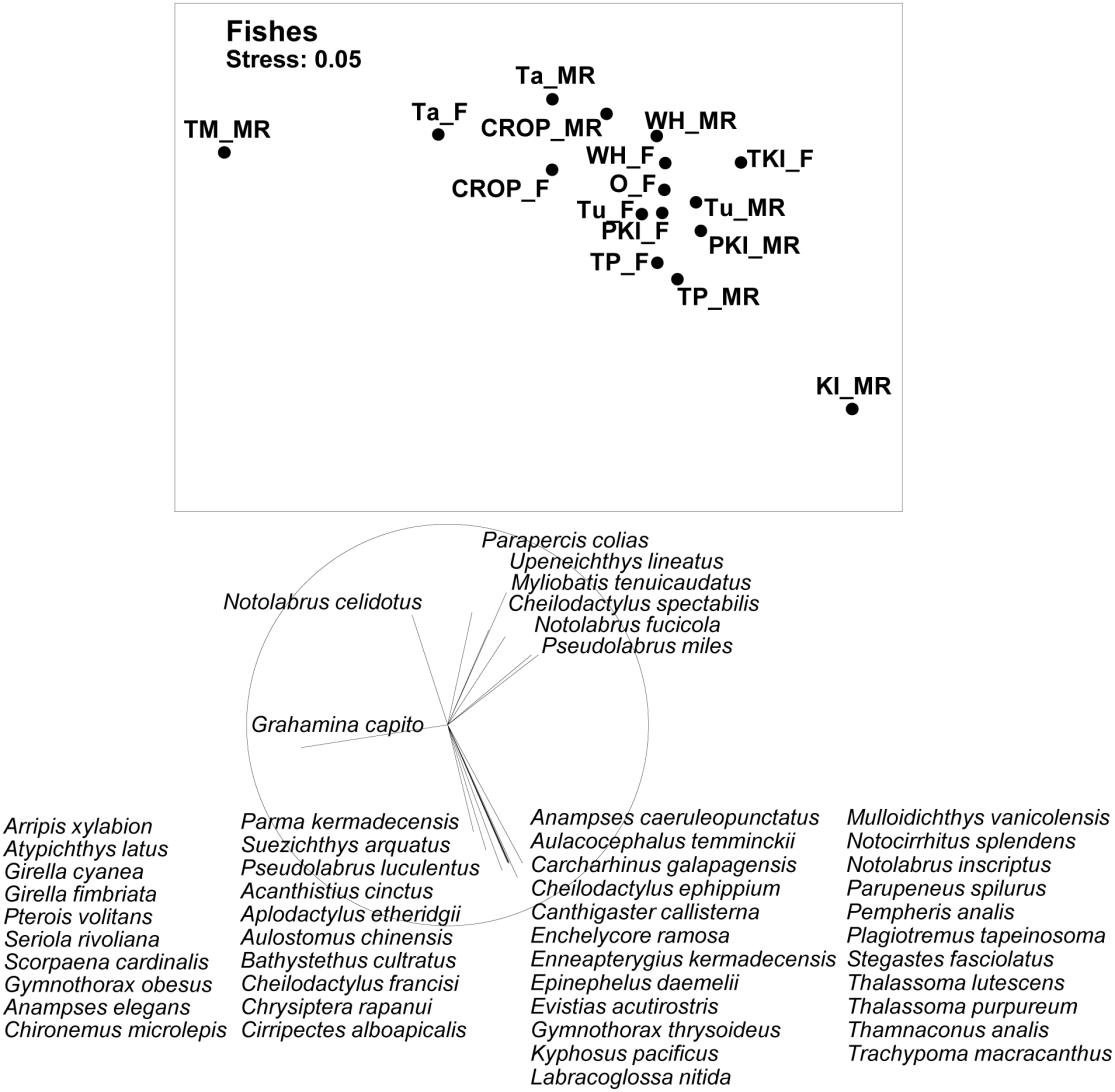
MDS plot of faunal relationships based on mean biomass of fish species at different sites in marine reserves (MR), fished reference sites adjacent to marine reserves (F), and fished sites at the Three Kings Islands (TKI_F) and around Northland (O_F). Marine reserves investigated are Cape Rodney-Okakari Point (CROP), Tawharanui (Ta), Whanganui a Hei (Wh), Te Matuku (TM), Poor Knights Islands (PKI), Kermadec Islands (KI) and Te Paepae o Aotea (TP). Vector plots are shown for fish species with high (>0.5) correlations with axes.

Assemblages of fishes observed along 5 m wide transects varied greatly between different marine reserve locations, with extreme outlier values at the Kermadec Islands and Te Matuku (Fig 2). The other six reserves within the MDS plot were positioned in close proximity to associated reference sites, indicating close faunal similarity; nevertheless, fish faunas outside marine reserves tended to group closely together overall, while faunas within marine reserves tended to be slightly outlying (Fig 2). The offshore marine reserves (Poor Knights Islands, Kermadec Islands, Tuhua and Te Paepae o Aotea) showed a consistent faunal shift to the bottom right of the plot when moving from nearby fished coast to reserve, while inshore reserves (Cape Rodney—Okakari Point, Tawharanui, Whanganui a Hei) trended to the top and right. Vector plots indicated a major separation between species associated with mainland northern New Zealand (7 species) and species associated with the Kermadec Islands plus offshore New Zealand reefs (43 species), while the triplefin *Grahamina capito* was associated with Te Matuku (Fig 2).

**Fig 3:**
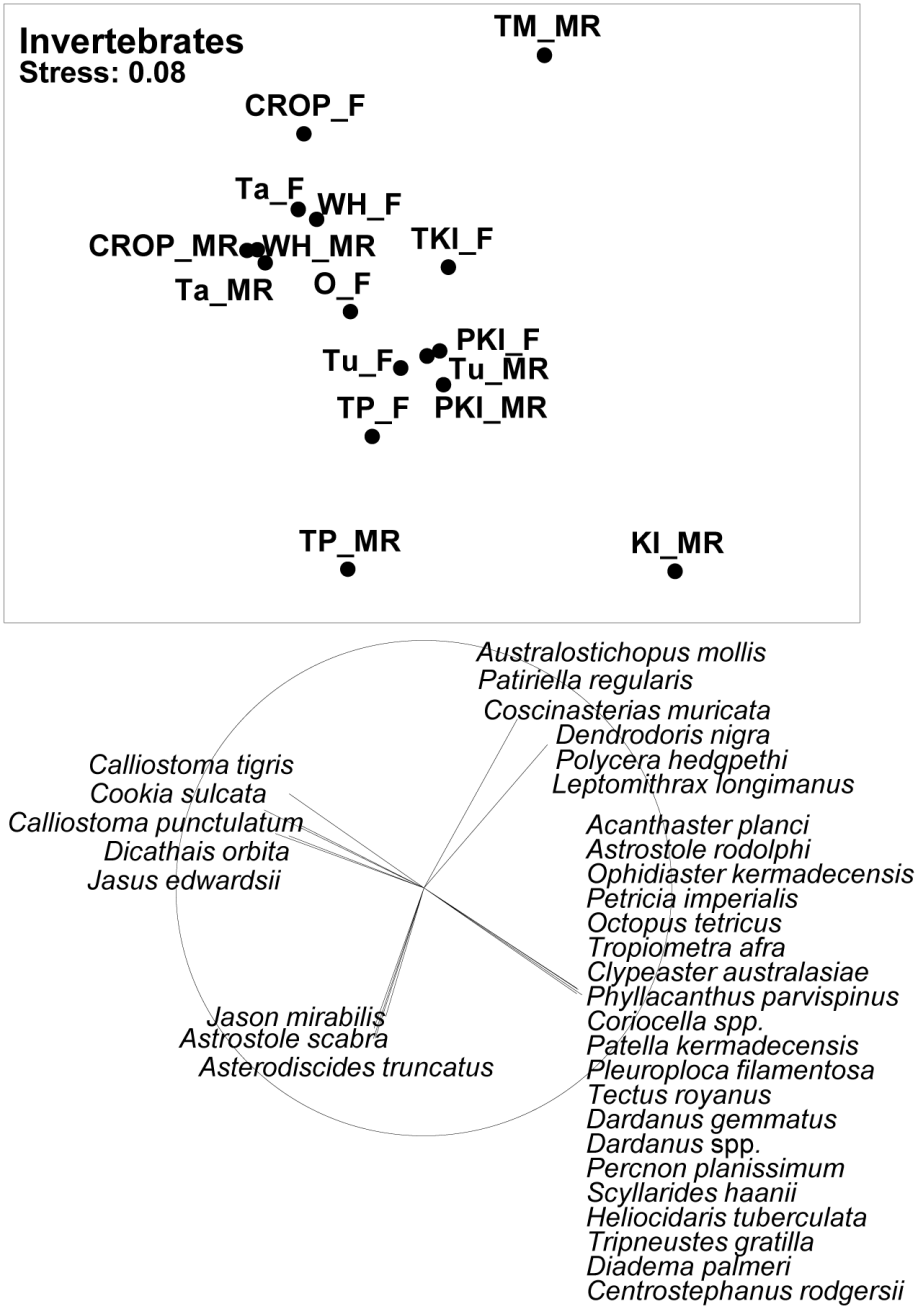
MDS plot of faunal relationships based on mean density of benthic invertebrate species at marine reserves (MR), fished reference sites adjacent to marine reserves (F), and fished sites at the Three Kings Islands (TKI_F) and around Northland (O_F). Marine reserves investigated are Cape Rodney-Okakari Point (CROP), Tawharanui (Ta), Whanganui o Hei (Wh), Te Matuku (TM), Poor Knights Islands (PKI), Kermadec Islands (KI) and Te Paepae o Aotea (TP). Vector plots are shown for invertebrate species with high (>0.5) correlations with axes.

As with fishes, invertebrate assemblages at Te Matuku and Kermadec Islands marine reserves were highly distinctive; coastal marine reserves tended to be below and to the left of associated fished coasts in the plot, and offshore marine reserves tended to be below and to the right of fished coasts (Fig 3). The invertebrate assemblage at the Three Kings Islands differed little from North Island assemblages. Four distinctive assemblages were evident in the invertebrate vector plot: (i) a coastal North Island assemblage that included the rock lobster *Jasus edwardsii* and topshell *Cookia sulcata*, (ii) an offshore North Island assemblage that included the seastar *Astrostole scabra*, (iii) a turbid inshore assemblage that included the seastar *Patiriella regularis* and the introduced nudibranch *Polycera hedgpethi*, and (iv) a large Kermadec Islands assemblage that included the seastar *Acanthaster planci* and urchin *Centrostephanus rodgersii*.

**Fig 4:**
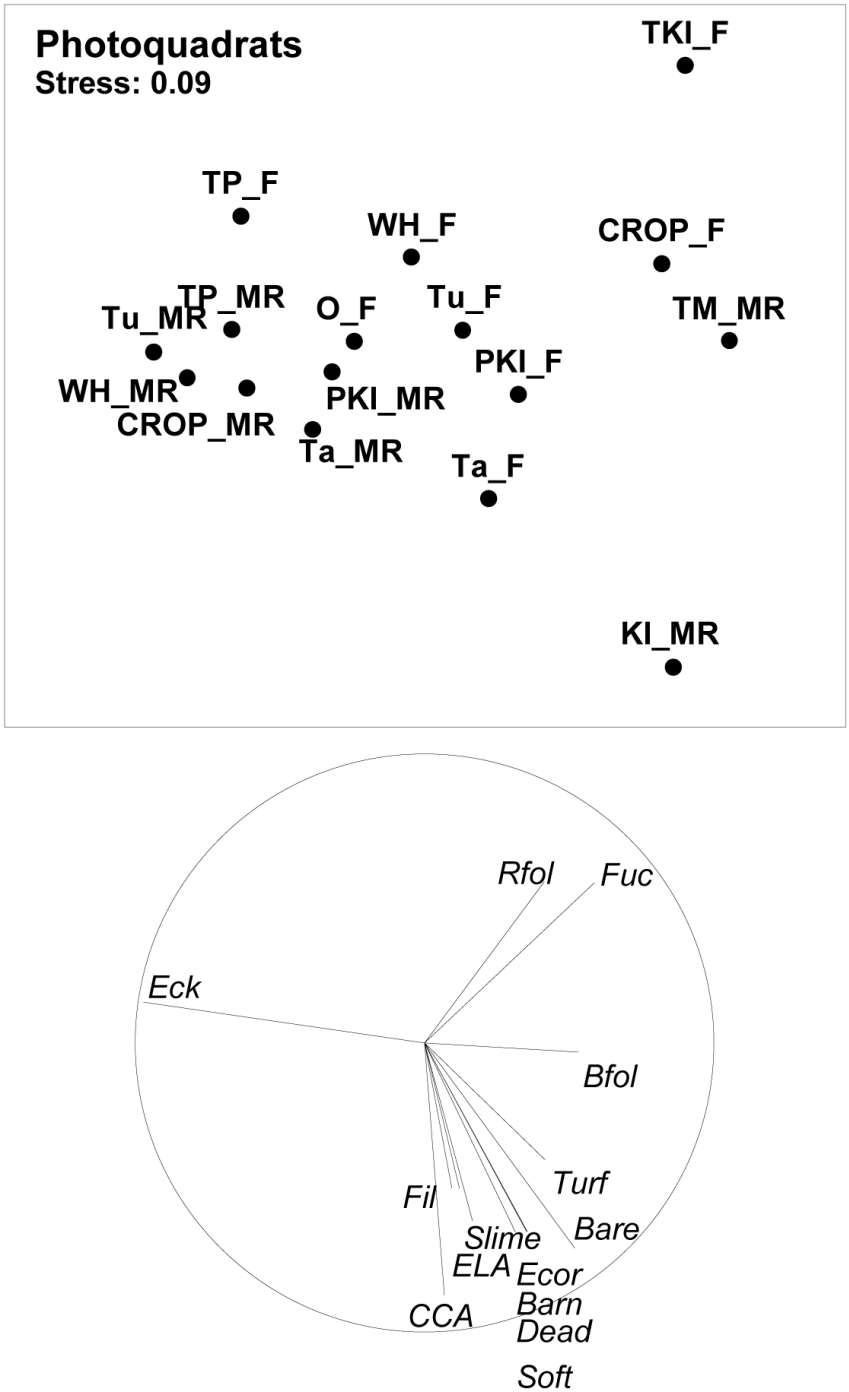
MDS plot of biotic relationships based on percent cover of different substrate cover categories at marine reserves (MR), fished reference sites adjacent to marine reserves (F), and fished sites at the Three Kings Islands (TKI_F) and around Northland (O_F). Marine reserves investigated are Cape Rodney—Okakari Point (CROP), Tawharanui (Ta), Whanganui o Hei (Wh), Te Matuku (TM), Poor Knights Islands (PKI), Kermadec Islands (KI) and Te Paepae o Aotea (TP). Vector plots are shown for taxa with high (>0.5) correlations with axes. Taxa abbreviations are explained in S1 Table.

On the basis of photoquadrat images, both the Kermadec and Three Kings Islands were found to possess habitat types distinctly different from North Island sites (Fig 4). Reefs at the Three Kings Islands tended to be dominated by red foliose algae and fucoid kelps, while substratum categories that were disproportionately represented at the Kermadec Islands included crustose coralline algae, encrusting leathery algae (e.g. *Peysonnelia*), bare rock, soft corals and encrusting stony corals.

In contrast to patterns evident for fishes and invertebrates, where North Island marine reserve locations tended to be more tightly clumped than fished locations, habitat types showed a greater range of variability at fished locations than reserve locations. North Island marine reserve locations other than Te Matuku were tightly clustered in the MDS plot (Fig 4), in large part because of a close association of *Ecklonia* with these reserve sites. When assessed quantitatively using the MvDisp multivariate index of dispersion, habitat types at sites within the six North Island marine reserves (excluding Te Matuku) showed greater homogeneity (0.98) than associated fished reference sites (1.14); whereas for fishes and benthic invertebrates the opposite patterns prevailed, with higher values in marine reserves (1.03 and 1.02, respectively) than associated fished sites (0.75 and 0.81, respectively).

### Differences between marine reserves and fished coasts

**Fig 5:**
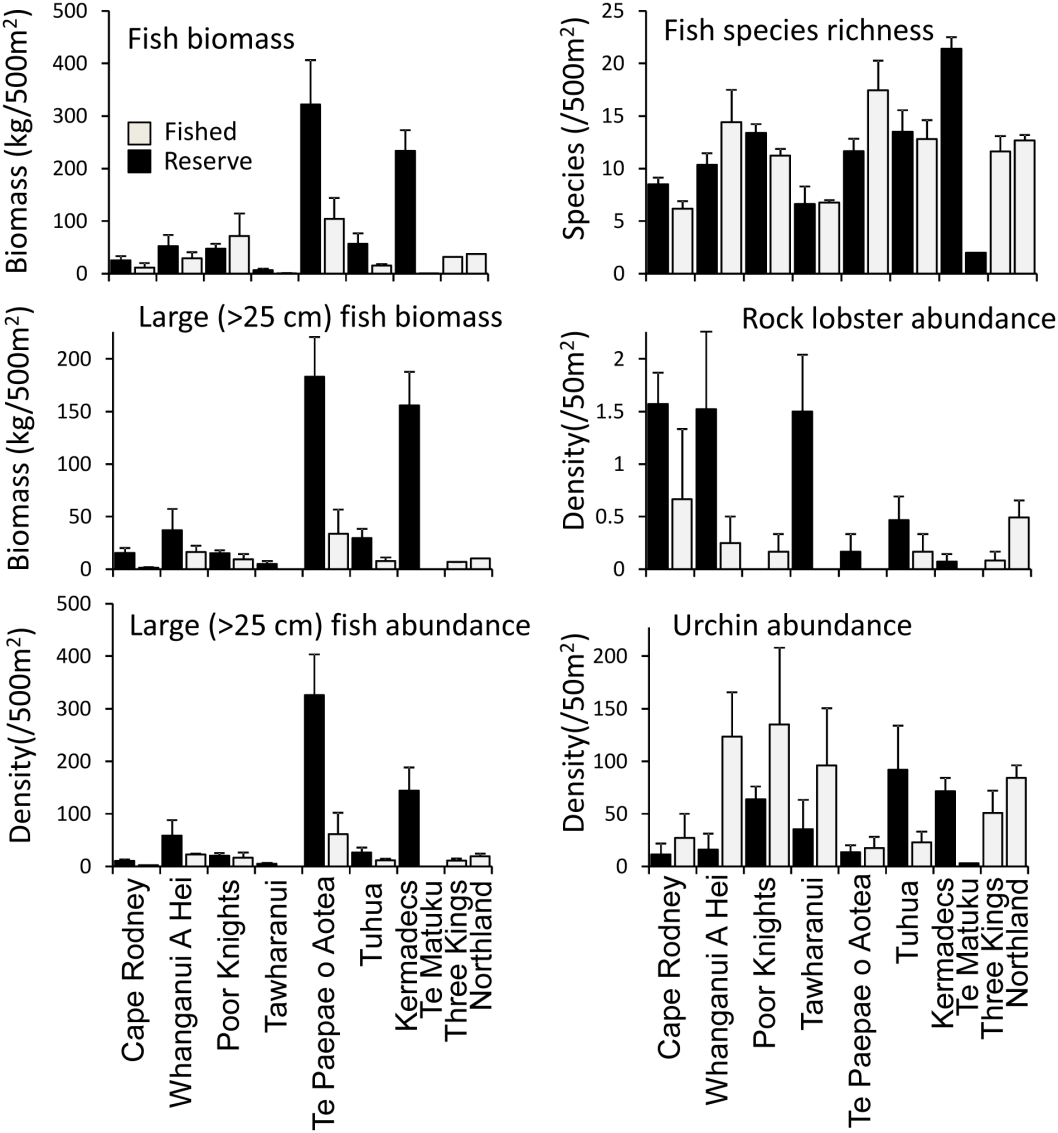
Means (± SE) of four fish community metrics and two invertebrate metrics in marine reserves, fished reference sites adjacent to marine reserves, and fished sites without associated marine reserves at the Three Kings Islands and around Northland. No fished reference sites were sampled near Kermadec Islands or Te Matuku. Note: scale of y-axis varies between panels.

Rocky reef communities protected inside marine reserves differed considerably among the reserves surveyed. The Kermadec Islands and Te Paepae o Aotea Marine Reserves had the highest fish biomass recorded, averaging ~220 and 330 kg fish biomass per 500 m^2^, respectively (Fig 5). At the Kermadecs, this biomass resulted from high densities of Galapagos sharks (*Carcharhinus galapagensis*), large schools of drummer (*Kyphosus sectatrix*), bluefish (*Girella cyanea*) and blue maomao (*Scorpis violacea*). Large kingfish (*Seriola lalandi*) were also common. In Te Paepae o Aotea, vast schools of blue maomao, two-spot demoiselles (*Chromis dispilus*) and pink maomao (*Caprodon longimanus*) were present at all three sites, along with numerous large blue moki (*Latridopsis ciliaris*) at one site (Small Volkner Rock). Te Matuku had the lowest average fish biomass; however, surveys at that site were compromised by poor visibility (Table 1), and fish data were not comparable to other surveys as a result. A consistent protection effect was evident across the six marine reserves with paired reference sites (*P* = 0.002, Table 3) when total fish biomass (log (x+1) transformed) was analysed using PERMANOVA.

**Table 3:** Results of univariate PERMANOVA for fish community metrics. Model design comprised two fixed factors: *location* (six reserves plus nearby reference sites) crossed with *zone* (inside/outside reserve). DF degrees of freedom, SS sum of squares, MS mean squares.

Source	df	SS	MS	Pseudo-F	P(perm)
Biomass					
Location	5	64.47	12.89	15.95	<0.001
Zone	1	8.81	8.81	10.90	0.002
Location x Zone	5	5.14	1.03	1.27	0.30
Error	45	36.38	0.81		
Total	56	107.63			
Large fish biomass					
Location	5	163.19	32.64	32.01	<0.001
Zone	1	57.31	57.31	56.20	<0.001
Location x Zone	5	58.58	11.72	11.49	<0.001
Error	45	45.89	1.02		
Total	56	265.39			
Fish abundance					
Location	5	64.55	12.91	12.47	<0.001
Zone	1	0.00	0.00	0.00	0.49
Location x Zone	5	1.01	0.20	0.19	0.97
Error	45	46.58	1.04		
Total	56	122.03			
Large fish abundance					
Location	5	53.76	10.75	16.05	<0.001
Zone	1	10.21	10.21	15.23	<0.001
Location x Zone	5	4.48	0.90	1.34	0.28
Error	45	30.15	0.67		
Total	56	93.21			
Fish species richness					
Location	5	2.92	0.58	8.98	<0.001
Zone	1	0.03	0.03	0.50	0.24
Location x Zone	5	0.52	0.10	1.59	0.19
Error	45	2.92	0.06		
Total	56	6.73			

Similar, but more exaggerated, patterns were evident when abundance and biomass of large (>25 cm length) fishes were considered (Fig 5; *P* <0.001, Table 3), although in this case significant variation in the ratio of large fish biomass inside versus outside reserves at different reserve locations manifest as a significant location x zone interaction (*P* <0.001). For all six marine reserves with associated fished reference sites, large fish abundance was higher inside the reserve compared to outside (*P* <0.001, Table 3), in most cases markedly so (Fig 5). By contrast, total fish abundance was not significantly higher inside reserves compared to outside (*P* = 0.49).

Fish species richness was greatest at the Kermadec Islands, which includes sub-tropical species. Species richness was also noticeably higher in reserves at offshore islands around the North Island than in coastal reserves (Fig 5). Some reserves showed higher mean fish species richness in the reserve compared to associated fished sites, while others showed the opposite trend. No consistent protection effect was detected using PERMANOVA (*P* = 0.24, Table 3).

**Fig 6:**
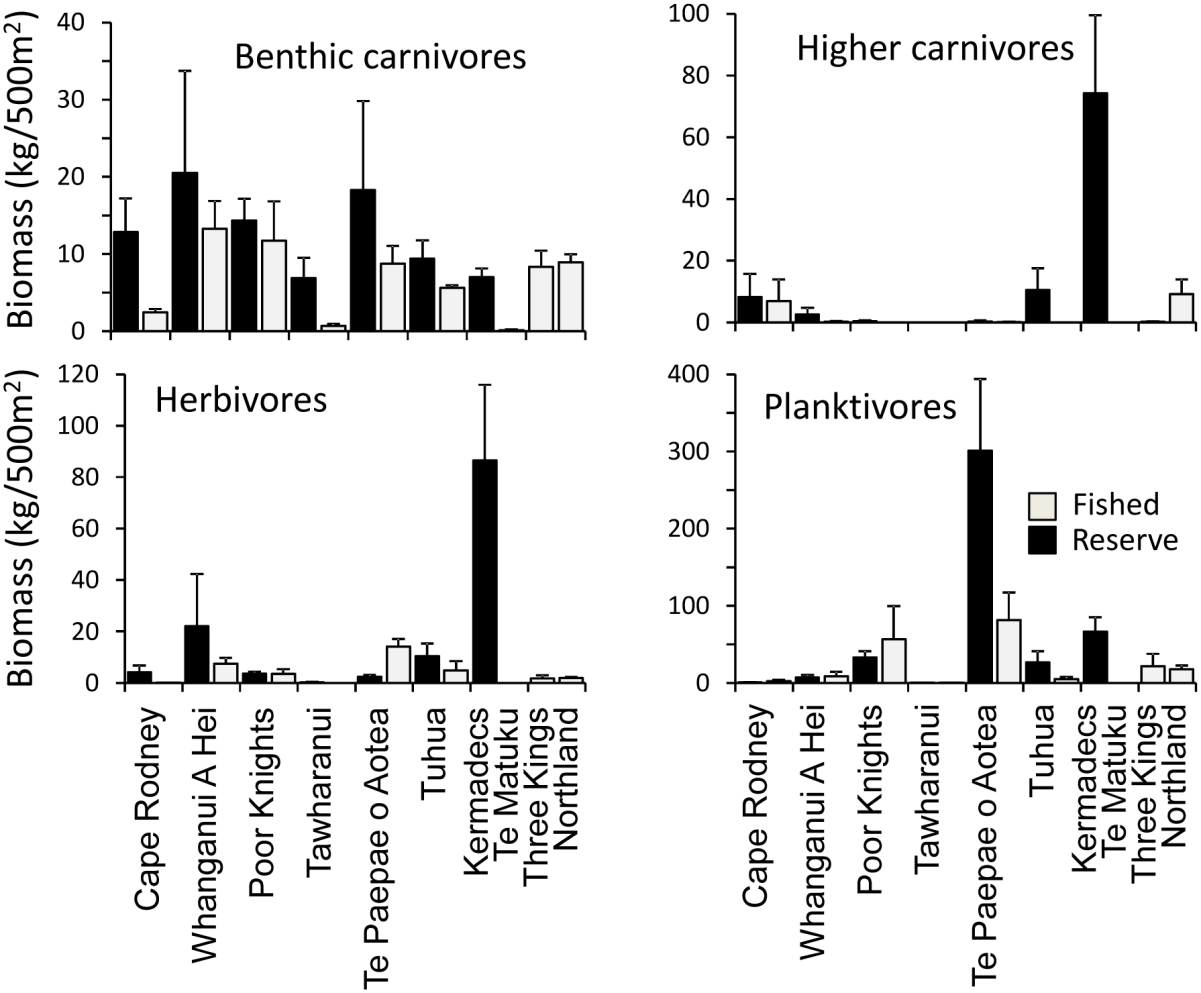
Mean biomass (± SE) of four major trophic groups for fishes observerd in marine reserves, fished reference sites adjacent to marine reserves, and fished sites at the Three Kings Islands and around Northland. Note: scale of y-axis varies between panels.

Significant (*P* < 0.05) reserve protection (zone) effects were evident for both benthic carnivores and higher carnivores, but not herbivores or planktivores, when the six reserves with associated fished sites were assessed using PERMANOVA (Table 4). For benthic and higher carnivores, biomass was higher in all reserves than associated reference sites (Fig 6). None of the four major trophic groups showed a significant zone x location interaction, whereas all but higher carnivores showed significant variation between locations. The lack of a significant location effect for higher carnivores was surprising given high apparent variability between reserves (Fig 6), however, variation between sites within locations was also extremely high.

**Table 4:** Results of univariate PERMANOVA for fish trophic metrics. Model design comprised two fixed factors: *location* crossed with *zone* (inside/outside reserve). DF degrees of freedom, SS sum of squares, MS mean squares.

Source	df	SS	MS	Pseudo-F	P(perm)
Benthic carnivore					
Location	5	18.44	3.69	4.47	0.006
Zone	1	5.66	5.66	6.87	0.007
Location x Zone	5	7.76	1.55	1.88	0.12
Error	45	37.09	0.82		
Total	56	62.68			
Herbivore					
Location	5	336.22	67.24	8.02	0.001
Zone	1	6.55	6.55	0.78	0.18
Location x Zone	5	31.32	6.26	0.75	0.57
Error	45	377.24	8.38		
Total	56	745.66			
Higher carnivore					
Location	5	116.41	23.28	1.56	0.18
Zone	1	50.01	50.01	3.35	0.039
Location x Zone	5	37.79	7.56	0.51	0.80
Error	45	672.63	14.95		
Total	56	923.27			
Planktivore					
Location	5	376.39	75.28	21.40	0.001
Zone	1	0.16	0.16	0.04	0.42
Location x Zone	5	11.47	2.29	0.65	0.63
Error	45	158.30	3.52		
Total	56	620.94			

**Fig 7:**
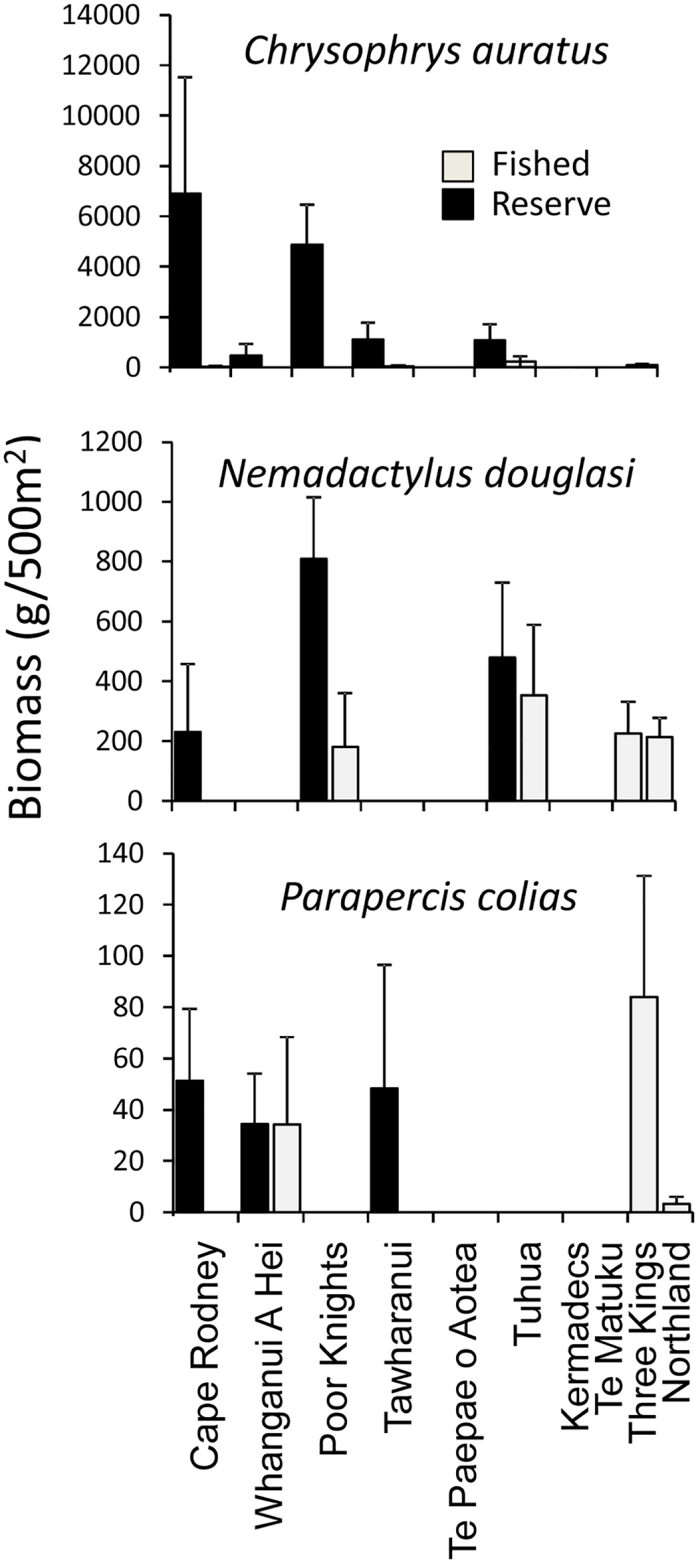
Mean biomass (± SE) of snapper *Chrysophrys auratus* in marine reserves surveyed, fished reference sites adjacent to marine reserves, and fished sites at the Three Kings Islands and around Northland.

Amongst individual fish species, the snapper *Chrysophrys auratus* showed significantly (*P* < 0.05) higher biomass relative to associated fished sites for the five reserves with associated reference sites where this species was recorded (Fig 7, Table 5). This species possessed a mean biomass of 2.7 kg per 500 m^2^ within the marine reserves and 0.07 kg at adjacent fished sites, a forty-fold difference. Two other targeted fishery species, porae *Nemadactylus douglasii* and blue cod *Parapercis colias* were also higher in reserves than adjacent reference sites, but low occurrence across reserves precluded statistical testing (Fig 7).

**Table 5:** Results of univariate PERMANOVA for common taxa. Model design comprised two fixed factors: *location* crossed with *zone* (inside/outside reserve). DF degrees of freedom, SS sum of squares, MS mean squares.

Source	df	SS	MS	Pseudo-F	P(perm)
*Chrysophrys auratus*					
Location	4	37.22	9.30	0.57	0.69
Zone	1	103.63	103.63	6.38	0.009
Location x Zone	4	27.26	6.81	0.42	0.78
Error	41	665.74	16.24		
Total	50	906.67			
Sea urchins					
Location	5	31.17	6.23	2.33	0.041
Zone	1	13.65	13.65	5.11	0.014
Location x Zone	5	16.39	3.28	1.23	0.29
Error	45	120.24	2.67		
Total	56	188.26			
Lobsters					
Location	5	2.08	0.42	4.00	0.010
Zone	1	1.22	1.22	11.77	0.003
Location x Zone	5	1.10	0.22	2.11	0.079
Error	45	4.67	0.10		
Total	56	11.43			

Although not sighted within the Poor Knights Island reserve, rock lobster abundance was significantly higher inside all other marine reserves when compared to associated fished sites (Fig 5; P = 0.003 in general test, Table 5). Mean rock lobster abundance was consistently observed to be ~ 1.5 animals per 50 m^2^ transect in the Cape Rodney-Okakari Point, Tawharanui and Whanganui a Hei reserves, while numbers in the other locations (including other marine reserves) were much lower (~0.3 per transect; Fig 5).

Sea urchin abundance also showed a consistent protection effect across the set of six reserves (*P* = 0.014, Table 5), with lower densities inside reserves than outside other than at Tuhua/Mayor Island. A low abundance of sea urchins is consistent with the hypothesis that relatively high lobster numbers in reserves reduce urchin densities. Within marine reserves, sea urchin abundance was highest at Tuhua/Mayor Island and the Poor Knights Islands due to high numbers of both *Evechinus chloroticus* and *Centrostephanus rodgersii*. Densities at these two reserves were similar to fished coastlines at ~80 per 50 m^2^ transect.

**Fig 8:**
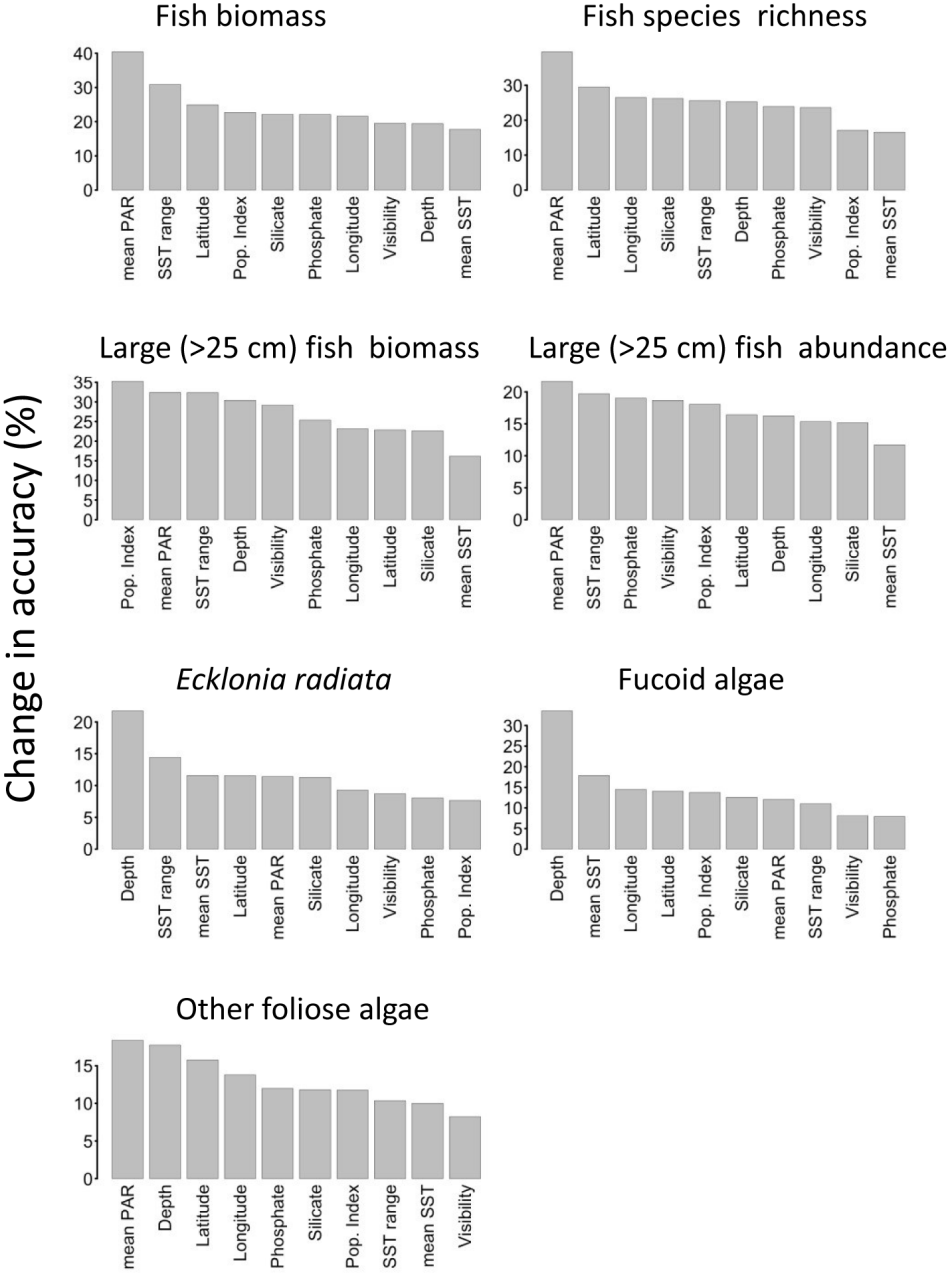
Relative importance of the 10 covariates used in prediction models developed with random forests. Note: scale of y-axis varies between panels.

The relative importance of different covariates to random forest models developed for different community metrics are shown in Fig 8. Mean photosynthetically-active radiation (BIO_parmean) was the most important covariate for predicting total fish biomass, total fish abundance, and fish species richness. However, the most important covariate for predicting large fish biomass was human population density, with the lowest large fish biomass values at sites near population centres. Annual sea surface temperature range (BIO_SST_range) was another important predictor for all fish metrics. Visibility was included as a predictor in random forest analyses, but was amongst the four most important predictors only for species richness of large fishes.

**Fig 9:**
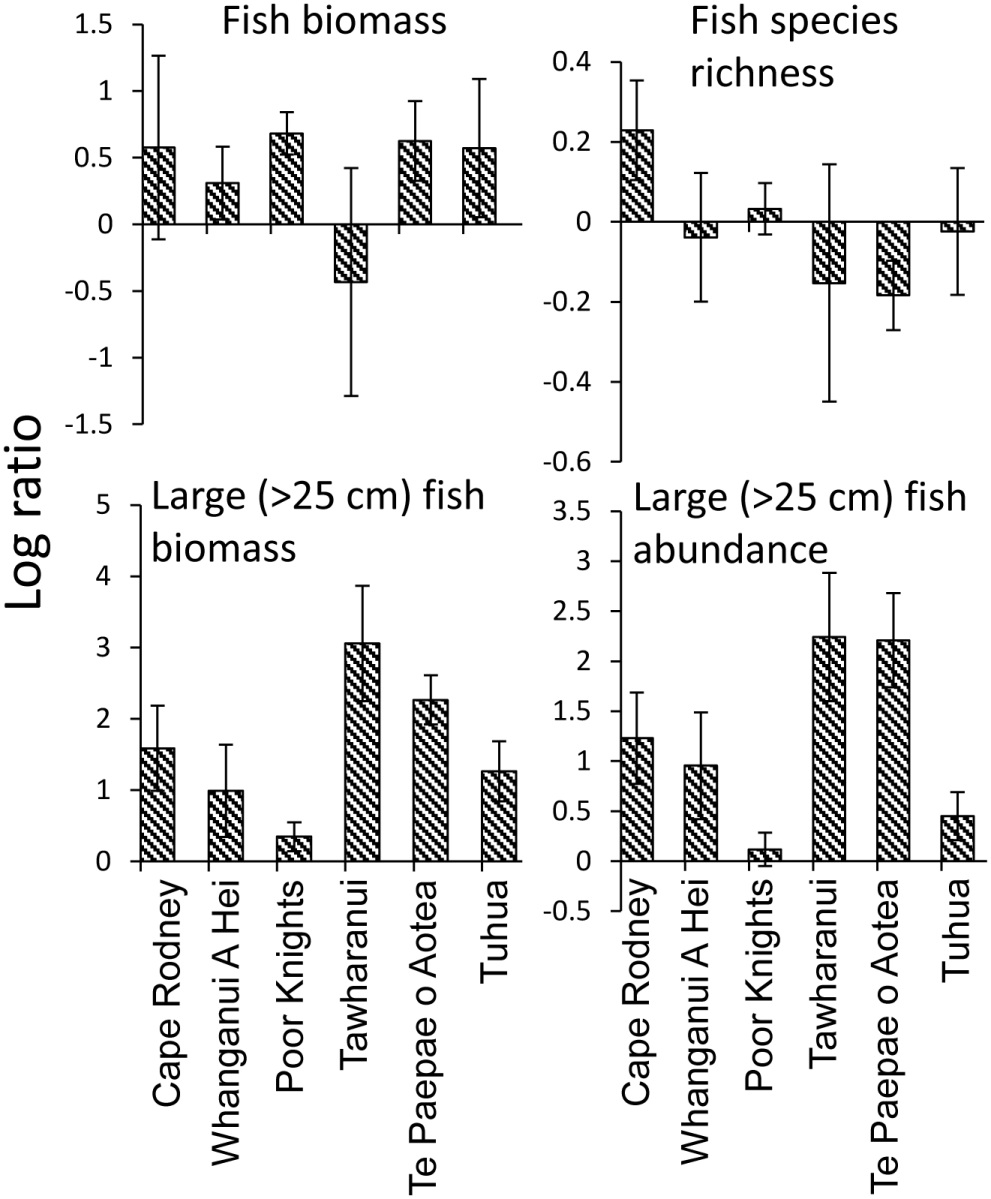
Effect size (± SE) for four fish community metrics at six marine reserves. Effect size was calculated using the log ratio (ln (observed)–ln (predicted)) where predictions were based on random forest relationships with 10 environmental covariates. Note: scale of y-axis varies between panels.

Outcomes of analyses where observed values were compared with random forest predictions agreed well with analyses based on nearby reference sites. All six marine reserves investigated using random forests had higher biomass and abundance of large fishes (> 25 cm) than predicted from models based on data from fished coasts (Fig 9). The mean log ratio for large fish biomass across the six reserves was 1.58 (a 388% increase). Fish species richness showed no consistent trend, while total fish biomass was disproportionately high in all reserves other than Tawharanui. Tawharanui Marine Reserve possessed few schools of fishes with small body size, and disproportionately many large individuals, generating the largest protection effect for large fish biomass

**Fig 10:**
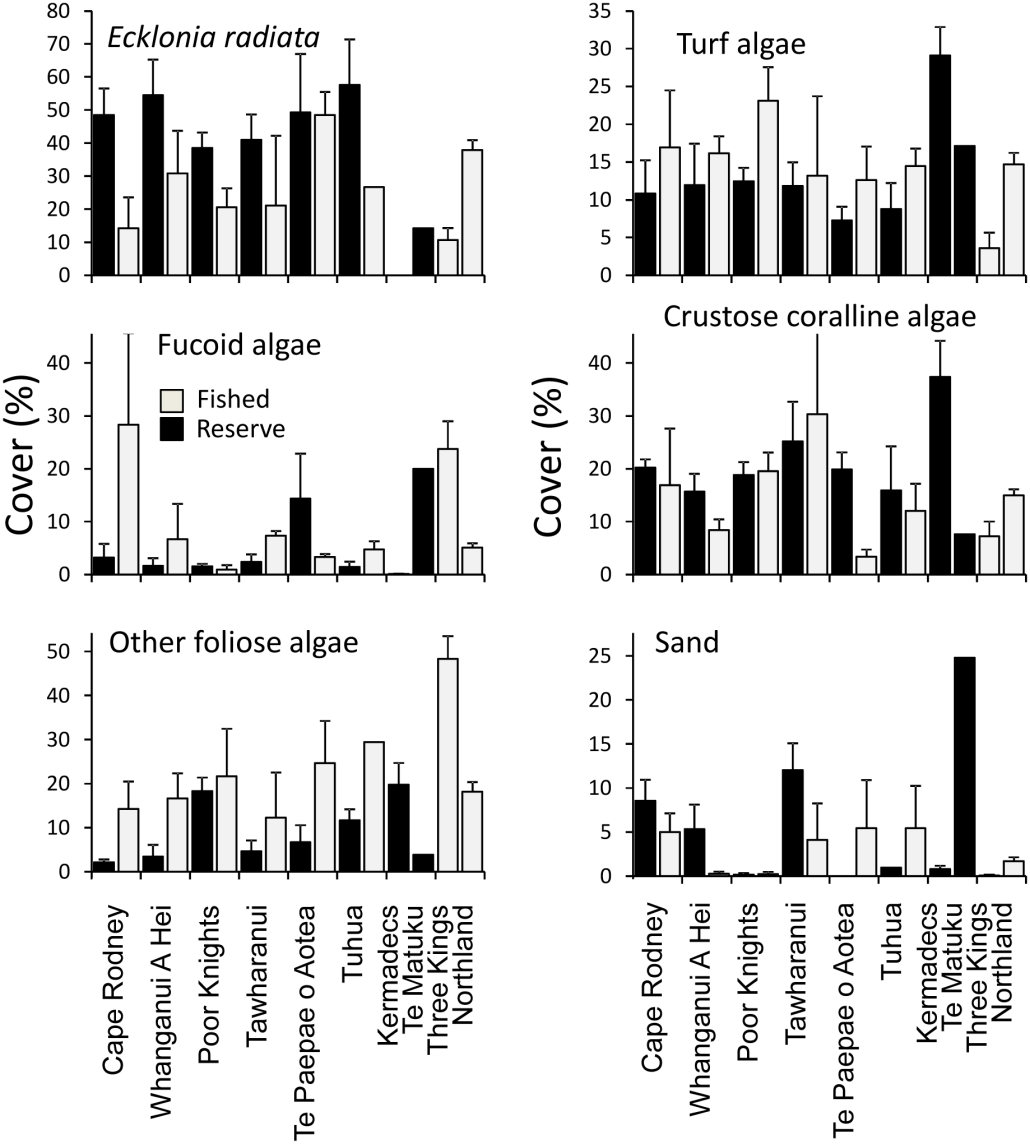
Mean cover (± SE) of different substratum categories in marine reserves, fished reference sites adjacent to marine reserves, and fished sites at the Three Kings Islands and around Northland. Note: scale of y-axis varies between panels.

Data from photoquadrats revealed strong consistencies in algal components across the marine reserves (Fig 10). All reserves possessed higher mean cover of *Ecklonia* than associated fished locations, with double the cover at Cape Rodney-Okakari Point, Tawharanui and Tuhua. This higher *Ecklonia* cover was found to be highly significant when assessed using PERMANOVA across the six marine reserves with fished reference sites (*P* = 0.005, Table 6).

**Table 6:** Results of univariate PERMANOVA for substrate metrics. Model design comprised two fixed factors: *location* crossed with *zone* (inside/outside reserve). DF degrees of freedom, SS sum of squares, MS mean squares.

Source	df	SS	MS	Pseudo-F	P(perm)
*Ecklonia radiata*					
Location	5	4.16	0.83	1.41	0.23
Zone	1	5.16	5.16	8.73	0.005
Location x Zone	5	3.04	0.61	1.03	0.39
Error	33	19.51	0.59		
Total	44	30.60			
Fucoid algae					
Location	5	8.10	1.62	1.88	0.14
Zone	1	3.94	3.94	4.58	0.018
Location x Zone	5	8.31	1.66	1.93	0.11
Error	33	28.44	0.86		
Total	44	48.58			
Foliose algae					
Location	5	7.89	1.58	2.12	0.080
Zone	1	10.21	10.21	13.71	0.002
Location x Zone	5	2.35	0.47	0.63	0.71
Error	33	24.56	0.74		
Total	44	52.04			
Turf algae					
Location	5	0.82	0.16	0.23	0.95
Zone	1	1.93	1.93	2.71	0.059
Location x Zone	5	0.95	0.19	0.27	0.92
Error	33	23.50	0.71		
Total	44	27.09			
Crustose coralline algae					
Location	5	3.44	0.69	1.54	0.222
Zone	1	1.11	1.11	2.49	0.058
Location x Zone	5	2.74	0.55	1.23	0.32
Error	33	14.74	0.45		
Total	44	21.98			

The total cover of fucoid algae and foliose algae also showed significant differences between reserves and fished sites, but in this case lower values were evident within reserves (Fig 10; *P* <0.05, Table 4). By contrast, turf algae and crustose coralline algae showed no significant protection effect overall (*P* >0.05, Table 4), albeit with statistical outcomes close to significance.

**Fig 11:**
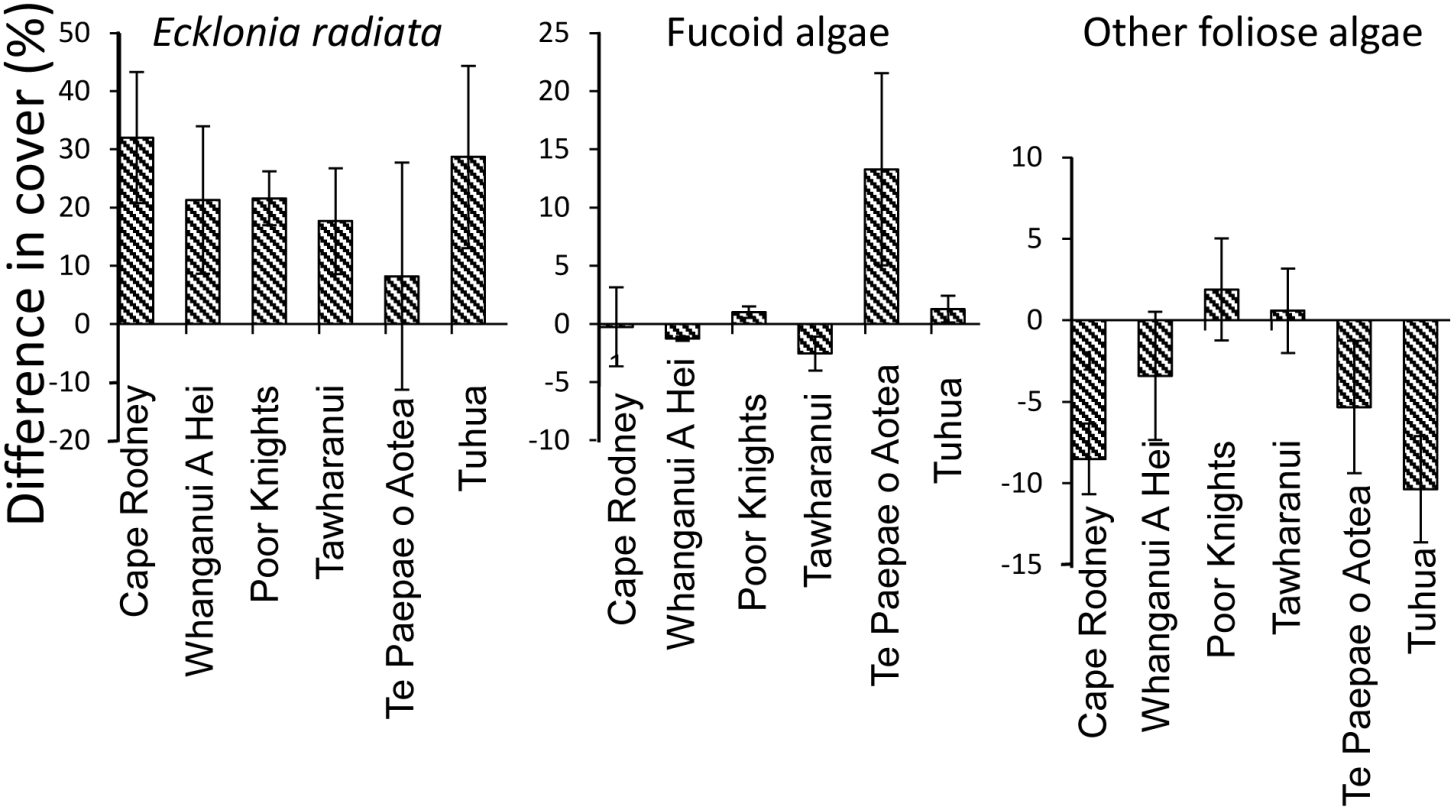
Effect size (± SE), as calculated using difference between observed and predicted values, for three algal cover metrics at six marine reserves. Predictions were based on random forest relationships with 10 environmental covariates. Note: scale of y-axis varies between panels.

The most important predictor of cover in random forest models for both *Ecklonia* and total fucoid algae was depth, while mean photosynthetically-active radiation was marginally more important than depth for the third photoquadrat metric with sufficient data for prediction using random forest models–‘other foliose algae’ (Fig 8). *Ecklonia* cover was ~20% higher at all marine reserves than predicted from models based on data on fished coasts (Fig 11). Fucoid algae showed a variable response, with very high levels at Te Paepae o Aotea marine reserve but little difference from predictions at other reserve locations. Other foliose algae tended to be lower than predictions, particularly at Cape Rodney-Okakari Point and Tuhua.

## Discussion

In line with expectations, significant protection effects were evident across North Island marine reserves that not only reflected direct interactions between fishing and targeted species (higher large fish biomass and lobster abundance) but also second order (lower urchin abundance), third order (higher *Ecklonia* cover) and fourth order (lower ‘other foliose’ and turf algal cover) interactions. While consistent with the conceptual model, the strength and regularity of observed differences between fished and unfished areas was surprising in that previous studies had indicated locally-idiosyncratic responses for different reserves [11]. Given that coincidental responses at six marine reserves distributed across a range of oceanographic conditions seems extremely unlikely, strong underlying ecological drivers associated with fishing were presumably operating.

Outcomes were not, however, all consistent with a simple top down trophic model, but included some unexpected results: (i) consistently higher (~20%) *Ecklonia* cover across North Island reserves relative to nearby fished sites regardless of lobster and urchin density, (ii) an inconsistent response of crustose coralline algae to urchin density, (iii) low cover of ‘other foliose’ and turf algae in marine reserves where urchin numbers were relatively low, and (iv) decoupling between responses of algal habitat to protection and responses of fishes and invertebrates to protection, as indicated by analysis of community heterogeneity across different sites. These results indicate considerable complexity in underlying mechanisms.

In line with the general conceptual model, a habitat-engineering role of urchins through grazing of macroalgae was supported by consistently low numbers of urchins at reserve sites with elevated cover of *Ecklonia*. The fished site with high *Ecklonia* cover, Te Paepae o Aotea, also had low urchin density. Nevertheless, an anomaly in this regard was Tuhua, where *Ecklonia* density was high within the reserve but urchin density was also high.

Urchin density was not, however, closely related to the extent of barrens. Crustose coralline algae, an important indicator of barrens extent in photoquadrats, possessed relatively consistent cover across the different North Island marine reserves, with no clear pattern inside versus outside reserves (Fig 10). Establishment and maintenance of urchin barrens is apparently a more complex process than simply reflecting high urchin density at the scale of whole transects, probably due in part to *Evechinus chloroticus* and *Centrostephanus rodgersii* exerting different levels of grazing pressure on northern New Zealand reefs, and to variability contributed by different urchin size classes. Moreover, different urchin density thresholds are known to occur for barrens formation than for barrens maintenance [27], meaning higher urchin densities prior to reserve protection may have left barrens patches that can be maintained by urchins at relatively low density. The degree of clumping/aggregation of urchins along a transect line may also play a role, with small dense aggregations of urchins able to create or maintain small barrens patches amongst macroalgal beds [28]. Nevertheless, as is the case with the extensive barrens in the Kermadec Islands [29], factors additional to urchins, such as excessive wave action and oceanographic conditions that restrict recruitment, may also limit cover of macroalgae in some situations.

Low densities of urchins inside reserves were associated with low densities of ‘other foliose algae’ and turf algae. This observation is contrary to the classical model of lobster eats urchins which graze macroalgae, in that lower urchin grazing within reserves is expected to lead to higher densities of all macro-algal types, not just *Ecklonia*. This outcome may partly result from the photoquadrat scoring process, whereby *Ecklonia* overlays other algae and so precludes scoring of those algae beneath. Nevertheless, few large algae, including ‘other foliose algae’, occur underneath *Ecklonia* due to insufficient light. Turf algae are obscured in photoquadrats by fucoid algae and other foliose algae as well as *Ecklonia*, which together consistently cover ~60% of the seabed. Cover estimates for turf algae could therefore be under-estimated by more than half, but without consistent bias associated with *Ecklonia*. Fourth order trophic interactions involving competition between algal species seems a more likely explanation, such that when grazing pressure is low within reserves then *Ecklonia* ultimately outcompetes other foliose and turf algae by developing a canopy that blocks light passing below. Experimental manipulative studies indicate that *Ecklonia* can inhibit development of turf algae in this way [30, 31].

The deviations from conceptual model predictions noted above for individual reserves could potentially result from low replication associated with those reserves, and a consequent high error in estimates of effect size. If, for example, more sites had been surveyed in Tuhua Marine Reserve, then a lower estimate of mean urchin density may have resulted, an outcome in line with predictions. Alternatively, Tuhua Marine Reserve may simply be anomalous compared to other locations with respect to urchin density and algal productivity, as also suggested in a prior study [32]. Regardless, the consistent elevation of *Ecklonia* cover within all reserves regardless of lobster and urchin density indicates major pathways linking biomass of fished species to macroalgae are probably operating.

Additional to rock lobsters, predatory fishes possibly contribute substantially to the control of herbivorous invertebrates in marine reserves, given that relatively few rock lobsters were observed in the offshore reserves (Poor Knights Islands, Tuhua and Te Paepae o Aotea reserves) where high cover of *Ecklonia* was present and overall levels of macro-algal herbivory presumably low. Large fishes were more than twice as abundant in these three reserves than random forest predictions. Consequently, fish species such as snapper, which partly feed on juvenile urchins when above 450 mm length [33], may contribute to control of urchins at reserve locations with few rock lobsters. An alternative explanation involving idiosyncratic combinations of local factors confounding analyses seems less likely given the consistency of the *Ecklonia* response across the reserves, and the use of offshore reference sites for comparisons involving offshore MPAs.

Herbivorous fishes potentially provide an important additional functional node in food webs. However, a strong linkage connecting fishing to herbivorous fishes has not been suggested for New Zealand waters to date, nor did the biomass of herbivorous fishes differ between reserves and associated reference sites (Table 4). Herbivorous fish species (most notably *Aplodactylus* spp., *Girella* spp., *Kyphosus* spp.) are rarely targeted by fishers in New Zealand, and generally possess large body size, so population numbers are unlikely to be greatly affected by elevated predatory fish numbers in reserves. Amphipods and other small grazing invertebrates (‘mesograzers’) probably play a greater functional role, as indicated by their high overall productivity and algal consumption rates [34, 35]. For example, amphipods have been implicated as an agent facilitating kelp dieback [36], and the amphipod *Orchomenella aahu* has been observed to destroy beds of *Ecklonia* stressed by bleaching or storm events [37]. Investigation of control of mesograzer populations by snapper and other large invertebrate-feeding fishes is urgently needed to better understand the dynamics of reef ecosystems [38].

Although data from the six primary reserves studied were generally consistent, these patterns rarely applied to the Kermadecs Islands or Te Matuku marine reserves (Fig 2), presumably because the former was biogeographically distinctive, and anomalously-turbid conditions prevailed at Te Matuku. No fished sites were located in comparable habitats for either reserve. Consequently, differences associated with marine reserve status could not be statistically assessed using either inside-outside contrasts or random forest modelling, which would have required model extrapolation rather than interpolation.

Macro-algal and invertebrate data from Te Matuku should not have been greatly biased by poor underwater visibility (<2 m) given the small area searched by divers and sedentary habits of target taxa. By contrast, fish data could be greatly affected by limitations associated with diver sighting so were not comparable to data obtained from other sites. Fish abundance estimates typically decline when visibility is less than 5 m (GJE, unpublished data). Visibility at Tawharanui Marine Reserve was also relatively low, so fish biomass values at that location were probably also biased downward relative to other reserves. Such a bias should not, however, have greatly affected comparisons with nearby reference sites, which had similarly low visibility.

A strong influence of light availability and water clarity on our ecological data also seems likely from the random forest modelling. Mean photosynthetically-available radiation (PAR), a remotely-sensed covariate with a high correlation with underwater visibility (r = 0.83), was the most influential covariate for four of the seven of the random forest models (Fig 8). PAR may provide a more useful indication of the light environment than visibility, as it is integrated across the year rather than recorded as a single measurement at the time of survey. Large fish biomass was most influenced by the human population index, presumably because of depletion of large fishes near population centres through fishing [39], while *Ecklonia radiata* and fucoid algae were primarily influenced by depth (negatively and positively, respectively).

Amongst the more intriguing outcomes of the study was the observation that the set of six northern New Zealand marine reserves tended to possess a more consistent algal habitat structure than the associated set of fished sites, whereas for fishes and benthic invertebrates the set of fished sites showed greater homogeneity in community structure than the set of reserve sites. Thus, protected reefs within reserves appear to have converged on a more uniformly *Ecklonia*-dominated habitat, while fishes and invertebrates did not track this habitat shift, but instead showed considerable differentiation from each other in the different reserves. The likelihood that the different patterns of homogeneity for fishes/invertebrates and macro-algae is real rather than an idiosyncratic artefact of local site variability is strengthened through analysis of floral and faunal data across the same set of sites, and strong relationships shown by fishes and invertebrates to variability in algal habitat in studies elsewhere [30, 40, 41].

The higher spatial heterogeneity in fish and benthic invertebrate communities within marine reserves compared to fished sites has important conservation implications that warrant further attention. If confirmed at broader scales, then it follows that the small proportion of the northeastern New Zealand biogeographic region (~0.2%) that is located within marine reserves plays a disproportionately large role in the national conservation of marine biodiversity, in that the reserves potentially include a greater range of fish and invertebrate communities than is present along the majority (>99%) of the North and South Island coasts that lies outside marine reserves.

Our investigation highlights the need for marine reserve investigations to encompass a wide range of ecosystem components rather than possess a narrow focus on exploited species directly affected by fishing. Surveys restricted to the large species targeted by fishers overlook other species with potentially fundamental ecological roles. Such groups as benthic invertebrates and macroalgae need greater research focus if trophic cascades [12, 42], and the full ecosystem consequences of fishing, are to be understood. The global marine reserve network comprises an irreplaceable manipulative experiment for improved understanding of the full magnitude of interactions between fishing and other threats, particularly climate change [43]. More should be made of this opportunity.

## Supporting information

S1 Table Substrate categories used for Reef Life Survey benthic photo-quadrat processing. (DOCX)
