# Supplementary material for: Consistent multi-level trophic effects of marine reserve protection across northern New Zealand
Source: PLoS One. 2017 May 24;12(5):e0177216. doi: 10.1371/journal.pone.0177216 (PMC5443496; doi:10.1371/journal.pone.0177216)
Supplement: S1 Table — (DOCX) [file pone.0177216.s001.docx]

S1 Table. Substrate categories used for Reef Life Survey benthic photo-quadrat processing.

| **Category code** | **Category name** |
| --- | --- |
| Aherm | Ahermatypic corals |
| Anem | Anemones and zoanthids |
| Asc | Ascidians |
| Bare | Bare rock |
| Barn | Barnacles |
| Bfol | Small to medium foliose brown algae |
| Bry | Bryozoan |
| Caul | *Caulerpa* spp. |
| CCA | Crustose coralline algae |
| Dead | Dead coral |
| Eck | Laminarian kelp, generally *Ecklonia radiata* |
| Ecor | Encrusting corals |
| ELA | Encrusting leathery algae |
| Fil | Filamentous epiphytic algae |
| FRA | Filamentous rock-attached algae |
| Fuc | Large brown fucoid kelps |
| GCA | Geniculate coralline algae |
| Gcal | Green calcified algae |
| Gfol | Other foliose green algae |
| Hyd | Hydroids |
| Peb | Pebbles/unconsolidated rocky bottom/coral rubble |
| Pol | Polychaete |
| Rfol | Foliose red algae |
| Sand | Sand |
| Slime | Diatom/algal/cyanobacterial fuzz/slime on bare rock |
| Soft | Soft corals and gorgonians |
| SpE | Sponges (encrusting) |
| Spo | Sponges (erect) |
| Stony | Large-polyp stony corals (e.g. fungiids) |
| Turf | Turfing algae (<2 cm high algal/sediment mat on rock) |
